# Dataset of proteomics analysis of aging *C. elegans* exposed to *Pseudomonas aeruginosa* strain PA01

**DOI:** 10.1016/j.dib.2017.02.001

**Published:** 2017-02-09

**Authors:** Christina D. King, Daljeet Singh, Kyle Holden, Annie B. Govan, Scott Keith, Arjumand Ghazi, Renã A.S. Robinson

**Affiliations:** a Department of Chemistry, University of Pittsburgh, Pittsburgh, PA 15260, USA; b Department of Pediatrics, Rangos Research Center, Children׳s Hospital of Pittsburgh, Pittsburgh, PA 15224, USA

**Keywords:** *C.elegans*, Proteomics, *Pseudomonas aeruginosa*, Aging, Immunity

## Abstract

Here, we present the proteomics dataset of young and middle-aged *Caenorhabditis elegans* (*C. elegans*) exposed to *Pseudomonas aeruginosa* (*P. aeruginosa* strain PA01), which is related to the article "Proteomic Identification of Virulence-Related Factors in Young and Aging *C. elegans* infected with *Pseudomonas aeruginosa*" (C. D. King et. al, in-revisions). This dataset was generated to better understand the effects of aging on molecular mechanisms involved in host response to pathogen exposure. Protein from *C. elegans* of different age and exposure to *P. aeruginosa* PA01 or control *E. coli* OP50 were extracted and tryptically digested. Peptides were labeled with the reagents tandem mass tag (TMT^6^-plex), separated, and detected by using offline strong-cation exchange and online liquid chromatography – mass spectrometry (SCX – LC – MS/MS & MS^3^). A separate mixture of peptides were labeled on N-terminal amines and lysines with dimethylation. Dimethylated peptides were analyzed using LC – MS/MS and a portion of the results were used to verify fold-change direction for TMT^6^-plex experiments. Raw data can be found online at www.CHORUSproject.org, a cloud-based data repository (see specifications table for details).

**Specifications Table**

**Table:** 

Subject area	Chemistry and Biology
More specific subject area	Aging, Proteomics, Immunity
Type of data	Mass spectra (RAW files), Proteome Discoverer (PD) readout files (Excel files), figures and table
How data was acquired	Offline Strong Cation Exchange (SCX) and online Liquid Chromatography – Mass Spectrometry (LC – MS/MS & MS^3^) or LC – MS/MS. LC parameters: NanoLC Ultra 2D (Eksigent) with AS-2 autosampler; 95 min gradient or 180 min gradient. MS parameters: LTQ Orbitrap Velos (Thermo Scientific), Top-ion selection (1-7 or 1-15) data dependent acquisition (DDA) method.
Data format	Raw mass spectra, un-filtered PD read-out files, and analyzed PD excel files
Experimental factors	Young (Day 1) and middle-aged (Day 5) *C. elegans* exposed to *P. aeruginosa* strain PA01 and aged-matched controls were used. *C. elegans* were grown to adulthood on *E. coli* OP50, then half were transferred to *P. aeruginosa* PA01 and exposed for 18 h. After pathogen exposure, *C. elegans* were harvested and homogenized, yielding protein homogenates for proteomics analysis.
Experimental features	Young (Day 1) and middle-aged (Day 5) *C. elegans* exposed to *P. aeruginosa* PA01, aged-matched controls, and a pooled sample containing equal amounts of the four samples were analyzed using proteomics techniques. Protein samples were digested with trypsin and resulting peptides were labeled by either TMT^6^ tags or dimethylation. TMT^6^-labeled and dimethylated peptides were subject to SCX-LC-MS/MS & MS^3^ or LC-MS/MS analysis, respectively.
Data source location	Pittsburgh, Pennsylvania, United States of America (USA)
Data accessibility	CHORUS: https://chorusproject.org/pages/dashboard.html#/projects/my/953/experiments
	Attached Supplemental document

**Value of the data**

• Mass spectra and resulting Excel files provide both qualitative and quantitative information about the aging proteome of *C. elegans* exposed to *P. aeruginosa* PA01.
• This data may be useful for individuals interested in comparing the proteome of *C. elegans* exposed to *P. aeruginosa* PA01 in comparison to various pathogens.
• This data extends the information available for proteome changes in aged-adult *C. elegans.*
• Data relating to changes in metabolism in middle-aged *C. elegans* exposed to *P. aeruginosa* PA01 may be used to gain further insight about the innate response of aging in higher-ordered organisms.

## 1 Data

**Fig. 1:**
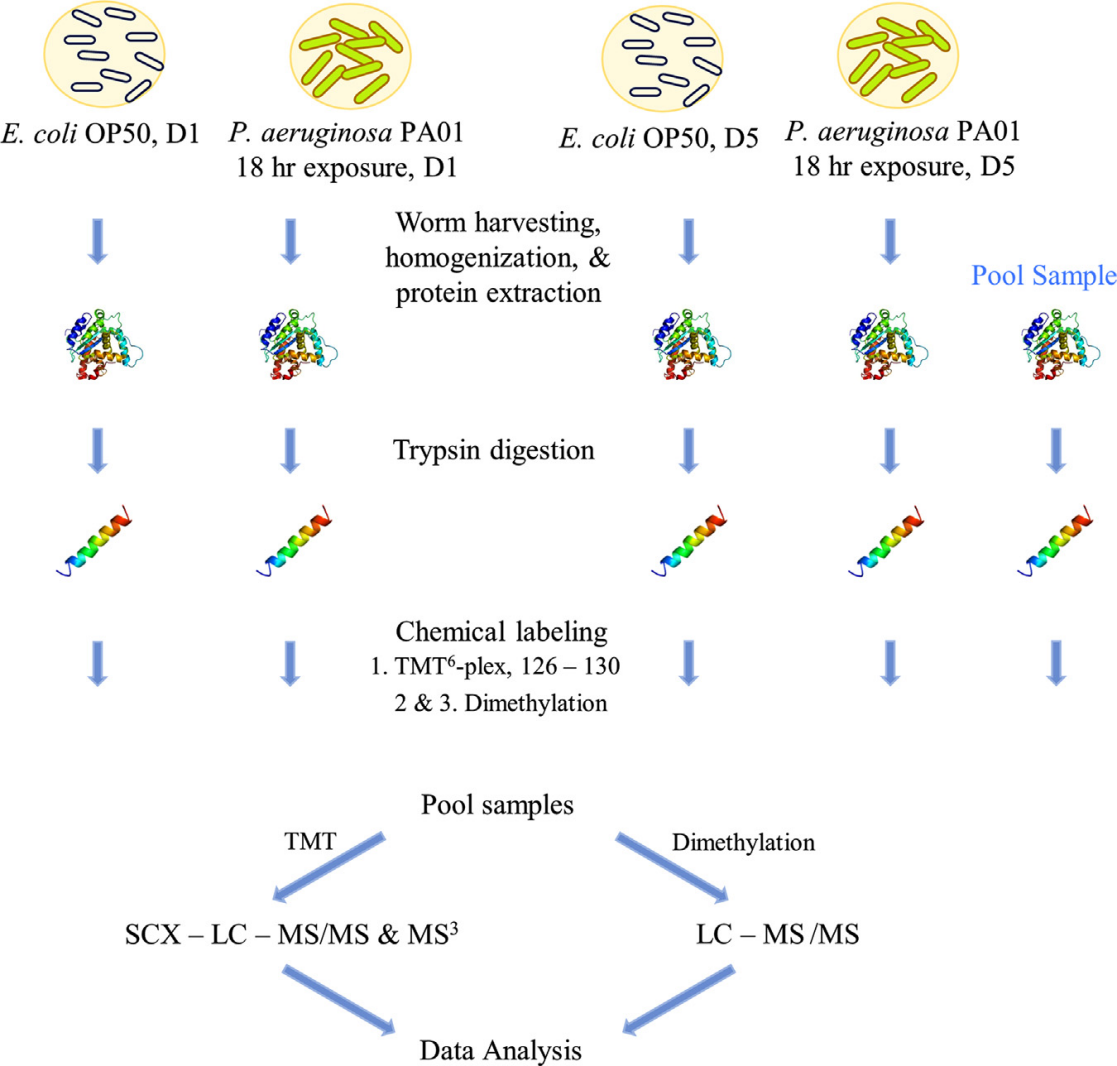
Sample preparation workflow of young and middle-aged *C. elegans* exposed to *P. aeruginosa* PA01 and aged-matched controls. *C. elegans* were exposed to *P. aeruginosa* PA01 for 18 h at either first (young) or fifth (middle-age) day of adulthood. Aged-matched controls were fed on *E. coli* OP50. After 18 h, worms were harvested, homogenized, and protein was extracted. A pooled sample containing equimolar amounts of the four groups was added. Protein was digested using trypsin and resulting peptides were chemically-labeled using either tandem mass tags (TMT^6^-plex) for the main analysis or dimethylation for the verification analysis. Labeled peptides were pooled, analyzed using LC – MS, and then analyzed using Proteome Discoverer v. 1.4.

**Fig. 2:**
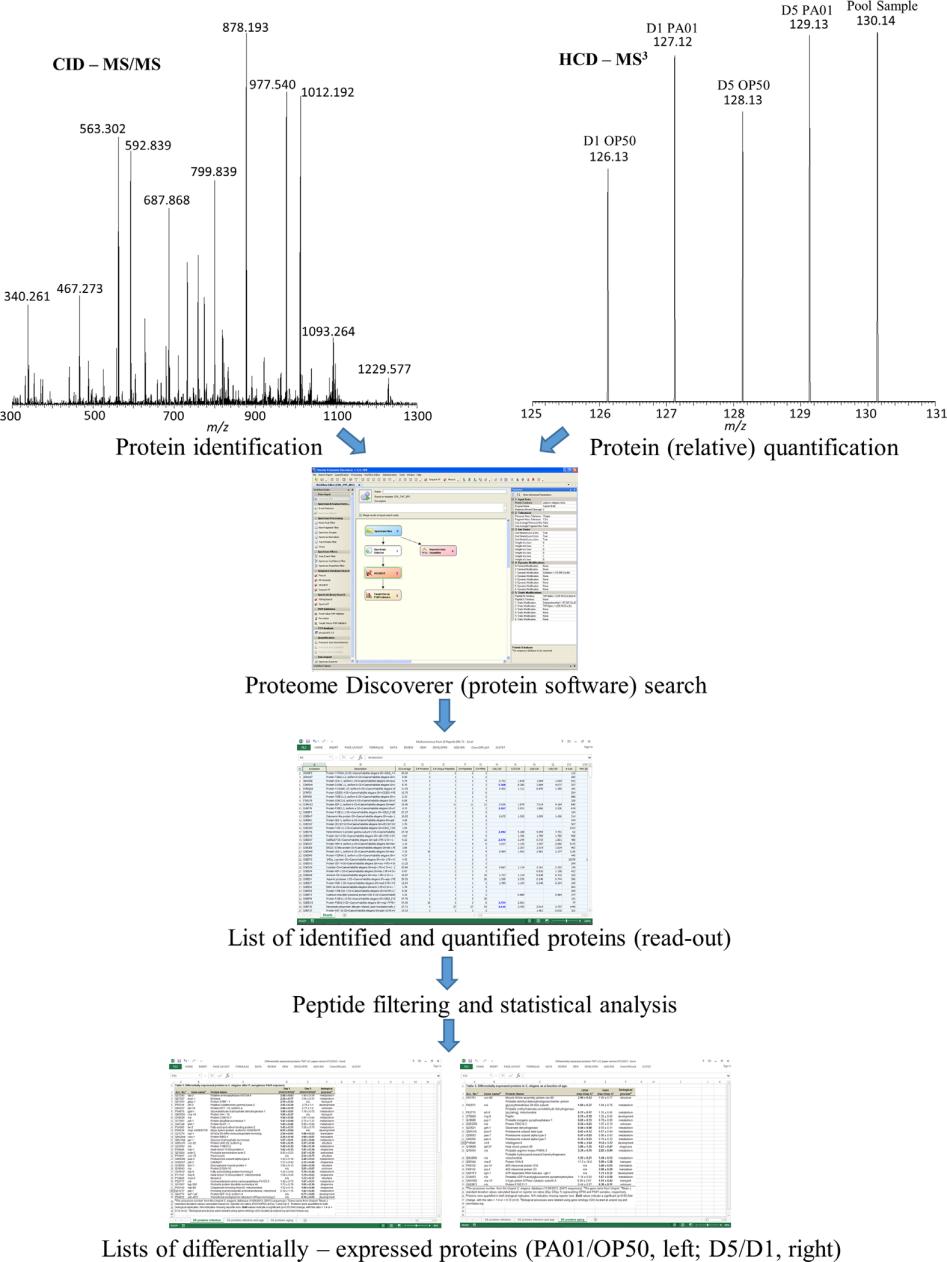
Data analysis workflow of aging *C. elegans* exposed to *P. aeruginosa* PA01. Tandem MS (MS/MS) and MS^3^ spectra were used to generate lists (per technical replicate) of identified and quantified proteins. Statistical analysis was applied to these lists. Proteins with a fold change ≥1.4 or ≤ 0.72 were determined to be statistically significant and added to a final list of differentially-expressed proteins.

Data presented here was generated by analyzing young and middle-aged *C. elegans* exposed to *P. aeruginosa* PA01, aged-matched controls, and a pooled sample containing equimolar amounts of all the samples. This dataset includes RAW mass spectra, Proteome Discoverer (PD) read-out files (Excel), and statistical analysis (Excel). RAW files generated were processed using PD, read-outs from the protein software were subjected to filtering and the Power Analysis statistical method [1], and final protein lists were searched using data analysis platforms (i.e PANTHERdb, Uniprot, and Wormbase). Fig. 1 displays the experimental workflow used to prepare and analyze samples and Fig. 2 explains the data analysis tools used to generate lists of differentially – expressed proteins. Table 1 explains the chemical-labels used for both sets of experiments.

## 2 Experimental design, materials, and methods

### 2.1 Sample preparation for proteomics analysis

Sodium hypochlorite L1 – synchronized worm populations were obtained and reared to early adulthood on *E. coli* OP50 – seeded NGM at 20 °C, (~80,000–100,000 total worms). To examine the proteome of young adults challenged with *P. aeruginosa*, one group of ~20,000 worms was transferred to *P. aeruginosa* PA01 – seeded SK plates on Day 1 of adulthood for 18 h after transfer to fresh *E. coli* OP50 – seeded NGM at 20 °C (again, transferring to fresh plates every other day to avoid progeny contamination). The second group was on *E. coli* OP50 – seeded NGM plates until Day 5 of adulthood before 18 h pathogen exposure and harvesting of both control and experimental groups. The parents were separated from eggs and progeny during transfers, and for harvesting, adults were washed off the plates with M9 and then allowed to settle by gravity. Two independent biological replicates were collected for both age groups to ensure reproducibility of important findings. At their respective time points, worms were floated off plates with M9 buffer [2] and washed two times to remove extraneous bacteria and obtain sample pellets.

### 2.2 Protein extraction

Worms were harvested, washed with M9 buffer to remove bacteria, and centrifuged to obtain a pellet. Pellets were re-suspended in Reassembly (RAB) buffer (0.1 M MES, 1 mM EGTA, 0.1 mM EDTA, 0.5 mM MgSO_4_, 0.75 M NaCl, 0.02 M NaF) with Roche Complete Protease Inhibitor (Roche Applied Science). Protein homogenate was sonicated for 10 s, 50 s on ice, incubated for 10 min on ice and centrifuged at 14000 g. The amount of protein was determined by BCA assay (Thermo Scientific). A pooled sample (Sample #5) containing equimolar ratios of Day 1 *E. coli* OP50 (Sample #1) and *P. aeruginosa* PA01 (Sample #2) and Day 5 *E. coli* OP50 (Sample #3) and *P. aeruginosa* PA01 (Sample #4) samples, was also prepared.

### 2.3 Protein digestion

Protein was purified using acetone precipitation and the amount of protein was re-determined with BCA assay. Protein (~80–100 µg) was denatured with an extraction buffer (0.2 M Tris, 8 M urea, 10 mM CaCl_2_, pH 8.0), reduced with 1:40 M excess of dithiothreitol (DTT) for 2 h at 37 °C, and then alkylated with 1:80 M excess of iodoacetamide (IAM) for 2 h on ice. The alkylation reaction was quenched by adding 1:40 M excess of cysteine and the mixture was incubated at room temperature for 30 min. Tris buffer (0.2 M Tris, 10 mM CaCl_2_, pH 8.0) was added to dilute the urea concentration to 2 M. Each sample was incubated with bovine TPCK-treated trypsin (Sigma–Aldrich) at 50:1 substrate/enzyme ratio for 24 h at 37 °C.

### 2.4 TMT labeling

Digested samples were desalted with an HLB cartridge and dried by centrifugal evaporation. Each sample was labeled with a TMT^6^-plex reagent following the manufacturer׳s protocol (Thermo Scientific). TMT^6^ reagents were equilibrated to room temperature, solubilized with 41 µL of acetonitrile, and transferred to peptide samples reconstituted in triethylammonium bicarbonate (TEAB) buffer. After 1 h of incubation (~25 °C), the reaction was quenched using 5% hydroxylamine. Equimolar amounts of samples were combined such that reagents that generate reporter ions at *m/z* 126:127:128:129:130 correspond to D1 OP50, D1 PA01, D5 OP50, D5 PA01, and the pooled sample, respectively.

### 2.5 Dimethylation labeling

Peptides (~50 μg) were reconstituted in 100 mM TEAB buffer (pH 8.5). The following solutions were added to *E. coli* OP50 samples for light (-CH_3_CH_3_) labeling and to *P. aeruginosa* PA01 samples for heavy (–^13^C^2^H_3_^13^C^2^H_3_) labeling: 4% formaldehyde (16 μL) and 0.6 M sodium cyanoborohydride (16 μL) (Sigma–Aldrich) or 4% ^13^C, D_2_ – formaldehyde (16 μL) and 0.6 M sodium cyanoborodeuteride (16 μL) (Sigma–Aldrich), respectively. Samples were vortexed for 10 min, quenched with 1% ammonia, and acidified with 5% formic acid. Samples were then desalted with an HLB cartridge, dried by centrifugal evaporation, and stored in the −80 °C freezer until further analysis.

### 2.6 Strong Cation Exchange (SCX) fractionation

SCX fractionation was performed on a PolySulfoethyl A 100 mmx2.1 mm, 5 µm, 200 Å column (The Nest Group, Inc.) with buffers as follows: mobile phase A was 5 mM monopotassium phosphate (25% v/v acetonitrile, pH 3.0), and mobile phase B was 5 mM monopotassium phosphate, 350 mM potassium chloride (25% v/v acetonitrile, pH 3.0). Dried TMT^6^– labeled samples were re-suspended in 200 µL of mobile phase A and injected onto the column. The gradient was as follows: 0–5 min, 0% B; 5–45 min, 0–40% B; 45–90 min, 40–80% B; 90–100 min, 80–100% B; 100–110 min, 100% B; 110–121 min, 0% B. Eluent was collected every minute and combined into 20 fractions. Each fraction was desalted using Supel-Tips C18 micropipette tips (Sigma–Aldrich). Fractions were solubilized in 50 µL and filtered with a 0.45 μm filter (Thermo Fisher Scientific).

### 2.7 LC–MS analyses

Online desalting and reversed-phase chromatography was performed with a Nano liquid chromatography (LC) system equipped with an autosampler (Eksigent). Mobile phases A and B used for reversed phase (RP)-LC separation of peptides were 3% (v/v) acetonitrile with 0.1% formic acid and 100% acetonitrile with 0.1% formic acid, respectively. SCX fractions (10 μL) were loaded onto a trapping column (100 µm i.d. x 2 cm), which was packed in house with C_18_ 200 Å stationary phase material (Michrom Bioresource Inc,) at 3 μL/min in 3% mobile phase B for 3 min. After desalting, the sample was loaded onto an analytical column (75 µm i.d. x 13.2 cm) which was packed in-house with C_18_ 100 Å 3 µm stationary phase material (Michrom Bioresource Inc). The gradient was as follows: 0–7 min, 10% mobile phase B; 7–67 min, 10–30% B; 67–75 min, 30–60% B; 75–77 min, 60–90% B; 77–82 min, 90% B; 82–83 min, 90–10% B; 83–95 min, 10% B. The LC eluent was analyzed with positive mode nanoflow electrospray using a LTQ Orbitrap Velos mass spectrometer (Thermo Fisher Scientific). Data-dependent acquisition parameters were as follows: the MS survey scan in the Orbitrap (300–1800 *m/z*) was 60,000 resolution; the top seven most intense peaks were isolated and fragmented with collision-induced dissociation (CID) in the LTQ (normalized collision energy of 35%). Directly after each tandem MS/MS scan, the most intense fragment ion over the m/z range 200–1545 was selected for higher-energy collisional dissociation (HCD) MS^3^. The fragment isolation width was set to 4 *m/z*, the MS^3^ AGC was 3×10^5^, the normalized collision energy was 60%, the resolution was 7,500 and the maximum ion time was 250 ms. HCD spectra were recorded in the Orbitrap. Each fraction was subject to duplicate injections.

Dimethylated samples were analyzed by LC – MS/MS. Similar mobile phases, trapping and analytical column settings, and instrument settings were used to perform the analysis, except the analytical gradient was: 0–5 min, 10% mobile phase B; 5–40 min, 5–40% B; 40–90 min, 15–25% B; 90–115 min, 25–30% B; 115–130 min, 30–60% B; 130–135 min, 60–80% B; 135–145 min, 80% B; 145–150 min, 80–10% B; 150–180 min, 10% B. Data-dependent acquisition parameters: MS survey scan in the Orbitrap (300–1800 *m/z*) with 60,000 resolution; the top fifteen most intense peaks were isolated and fragmented with CID in the LTQ (normalized collision energy of 35%). Each fraction was also subjected to duplicate injections (technical replicates).

### 2.8 Data analyses

RAW files were analyzed with Proteome Discoverer 1.4 software (Thermo Scientific) and searched against the Uniprot *C. elegans* database (11/26/2013, 25,673 sequences). SEQUEST search parameters were as follows: two maximum trypsin mis-cleavages, precursor mass tolerance of 10 ppm, fragment mass tolerance of 0.8 Da; static modifications were TMT six-plex/+229.163 Da (N-terminus, Lys) and carbamidomethyl modification/+57.021 Da (Cys); dynamic modification was oxidation modification/+15.995 Da (Met). Decoy database searching was employed to generate medium (*p*<0.05) and high (*p*<0.01) confidence peptide lists. All the peptides with medium and high confidence were used to identify and quantify proteins. To filter peptides, the following parameters were applied: peptide confidence level of medium or high, peptide rank of 1, and peptide deviation of 10 ppm. Peptides with a PSM (peptide to spectral match) count of 1 (per technical replicate) were not considered for analysis. The reporter ions (i.e. *m/z* 126–130) were identified with the following parameters: most confident centroid and 20 ppm for reporter ion mass tolerance. Furthermore, reporter ion values were normalized 126/130, 127/130, 128/130, and 129/130 and final ratio reporting given as 127/126 and 129/128. Proteins belonging to multiple isoforms were grouped into a single accession number and final ratios were reported.

### 2.9 Statistics

Coefficient of variation (CV) values were calculated as previously explained for reporter ion ratios of proteins quantified in both biological replicates [1]. The mean CV value from both biological replicates was calculated and used as the total biological variation, *S*_*b*_*,* (i.e. 0.36). The technical variation, *S*_*t*_, was calculated for proteins quantified in at least one technical replicate of each biological replicate, and was 0.22. This power analysis was used to generate an appropriate fold-change cutoff for TMT^6^-plex data. Filter criteria were applied to generate a list of statistically significant differentially-expressed proteins as follows: 1) proteins identified and quantified in at least one technical replicate (per biological replicate), 2) CV values ≤0.36, and 3) fold-change cutoff dependent upon n as such ≥1.4 or ≤0.72 (*n*=2).

Data from the dimethylation experiment was treated by applying the following criteria: 1) proteins identified and quantified in both technical replicates and 2) fold-change cutoff of ≥1.4 or ≤0.70 dependent on *p*<0.05.

## Figures and Tables

**Table 1:** Chemical – labeling strategy for proteomics analysis of aging *C. elegans* exposed to *P. aeruginosa* PA01.

	**D1 OP50**	**D1 PA01**	**D5 OP50**	**D5 PA01**	**Pool sample**
**TMT**^**6**^**-plex**	126	127	128	129	130
**Dimethylation**	Light	Heavy	Light	Heavy	n/a
