# Supplementary material for: Dataset of proteomics analysis of aging C. elegans exposed to Pseudomonas aeruginosa strain PA01
Source: Data Brief. 2017 Feb 9;11:245–51. doi: 10.1016/j.dib.2017.02.001 (PMC5320047; doi:10.1016/j.dib.2017.02.001)
Supplement: Supplementary file 1 — Supplementary material [file mmc1.docx]

**Conflict of Interest**

The authors have no conflicts of interests to report.
